# Sulfasalazine mitigates cisplatin-induced nephrotoxicity via NF-κB downregulation and upstream modulation of p-AMPK, SIRT1, miRNA-132-3p, and miRNA-375

**DOI:** 10.1007/s00210-026-05009-1

**Published:** 2026-02-18

**Authors:** Mohammed G. Hassan, Ihab T. Abdel-Raheem, Asser I. Ghoneim, Maged W. Helmy

**Affiliations:** 1 Pharmacology and Toxicology Department, Faculty of Pharmacy, Damanhour University, Damanhour, El-Bahira, Egypt; 2 Department of Clinical Pharmacy & Pharmacy Practice, Faculty of Pharmacy and Drug Manufacturing, Pharos University in Alexandria, Canal El Mahmoudia Street, beside Green Plaza Complex, Alexandria, Egypt; 3 Pharmacology and Toxicology Department, Faculty of Pharmacy, Damanhour National University, El-Bahira, Egypt

**Keywords:** Cisplatin, Sulfasalazine, Nephrotoxicity, NF-κB-p65, MiRNA

## Abstract

The present study tested for the first time the safety of sulfasalazine at dose of 100 mg/kg on renal function, in addition to study its possible renal effects on cisplatin-induced nephrotoxicity in rat model. Furthermore, it aimed to investigate the modulatory effect of sulfasalazine on NF-κB. Forty-eight male Sprague–Dawley rats were divided into four groups: negative control (given a daily oral dose of normal saline), cisplatin (given a single dose of Cisplatin, 6 mg/kg, i.p.; nephrotoxicity model), sulfasalazine (given a daily dose of SFZ, 100 mg/kg, orally via gavage; control model), and Cis + SFZ (given 100 mg/kg sulfasalazine, orally, 5 days before and 14 days after the cisplatin dose). Nephrotoxicity was evaluated 96 h and 14 days following cisplatin administration. Cisplatin nephrotoxicity was evidenced by significant rises in renal indices of functional, inflammatory, fibrotic, apoptotic and histological profiles. It also up-regulated miR-375 and miR-132-3p expression. Decreased p-AMPK, SIRT-1, HNF-1B and total antioxidant levels in kidney homogenates were also observed at 96 h following cisplatin injection. Sulfasalazine alone didn’t show any change in renal parameters compared to the saline-treated group but counteracted cisplatin-induced nephrotoxic changes. The obtained results revealed that sulfasalazine at the dose of 100 mg/kg offered a renoprotective effects against cisplatin-induced nephrotoxicity which could be attributed to its inhibition of NF-κB-p65 via downstream attenuation of inflammatory, apoptotic and fibrotic pathway. This inhibitory effect of sulfasalazine on NF-κB-p65 could be at least in part attributed to its upstream modulation of p-AMPK, SIRT1, miRNA-132-3p and miR-375.

**Graphical abstract:**

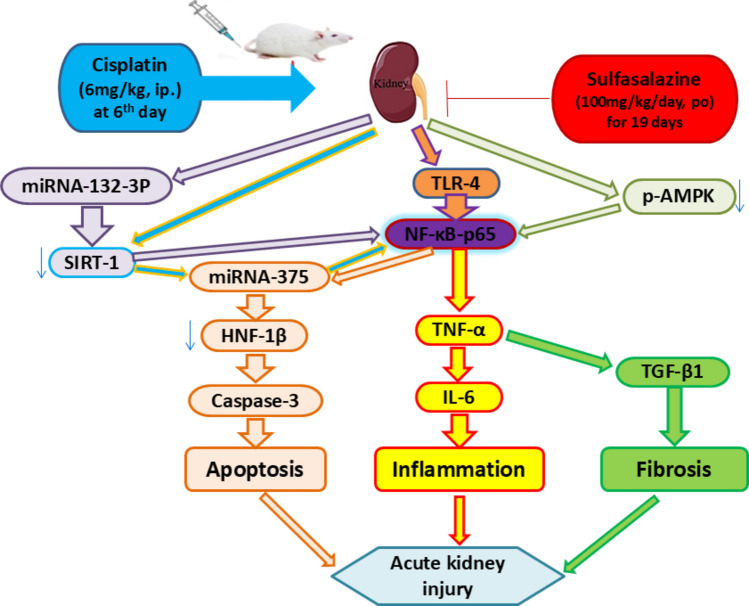

**Supplementary Information:**

The online version contains supplementary material available at 10.1007/s00210-026-05009-1.

## Introduction

Cisplatin is a cytotoxic agent used to treat a variety of tumor types. However, its dose-limiting nephrotoxicity is a significant drawback that limits the drug’s use and becomes an urgent issue that needs to be solved. The pathophysiology of cisplatin-induced nephrotoxicity mainly includes inflammation, fibrosis, apoptosis and oxidative stress (Loren et al. 2021; Tolouian et al*.*^2023^).

An important part of the pathophysiology of cisplatin-induced nephrotoxicity is inflammation. Recent research has focused on the inflammatory pathways associated with cisplatin-induced nephrotoxicity, such as the inflammatory response driven by nuclear factor-kappa B (NF-κB) (Altındağ 2022). NF-κB is a crucial transcription factor in eukaryotic cells that controls gene expression during inflammatory and immune responses. It has been discovered that NF-κB is a promising target for reducing the inflammatory response in a variety of diseases (Du et al*.*^2019^). Within the nucleus, cisplatin-induced activation of NF-κB promotes transcription of inflammatory mediators like tumor necrosis factor alpha (TNF-α) and other inflammatory cytokines (IL-1, IL-6, etc.), ultimately leading to cisplatin-induced nephrotoxicity (Zhao and Wen 2018).

There are different crosstalk signaling pathways involved in NF-κB-driven cisplain-induced nephrotoxicity. First, TLR-4/NF-κB/TNF-α/IL-6 crosstalk signaling pathway. In this pathway, cisplatin induces release of endogenous TLR-4 ligands such as damage associated molecular patterns (DAMPs), in addition to NF-κB induction, which resulting in enhanced inflammatory cytokines production in a TNF-α dependent manner, further contributing to the development of renal inflammation (Manohar and  and Leung 2018). Second, TGF-β1/TNF-α signaling pathway plays a critical role in long term renal fibrosis, further promoted TNF-α driven renal injury induced by cisplatin (Volarevic et al. 2019).

Moreover, Hao et al. demonstrated that cisplatin mediates tubular cell apoptosis through induction of NF-κB and hence, induction of miRNA-375 with subsequent repression of the nephroprotective protein HNF-1B (Hao et al. 2017). Similarly, NF-κB induction by cisplatin could be correlated to its targeting of miRNA-132-3p/SIRT-1 pathway which mediates apoptotic and inflammatory responses of renal tubular epithelial cells (Han et al. 2021). In addition, the suppression of p-AMPK/SIRT-1 pathway potentiates NF-κB nuclear translocation and increased the expression level of NF-κB-driven renal inflammatory response (TNF-α and IL-6) (Du et al*.*^2019^; Han et al. 2021; Sung et al. 2020). Therefore, the transcription factor NF-κB plays a key role in the pathogenesis of cisplatin-induced nephrotoxicity and hence, attenuation of NF-κB–driven signaling pathways through pharmacological and genetic approaches is an urgent issue to prevent cisplatin-induced nephrotoxicity (Humanes et al. 2017).

Sulfasalazine is a disease-modifying antirheumatic drug (DMARD) used in the management of autoimmune conditions such as rheumatoid arthritis and inflammatory bowel disease through different mechanisms including NF-κB inhibition (Cámara-Lemarroy et al. 2009; Demirbilek et al. 2007), that could be a promising avenue for amelioration of cisplatin-induced nephrotoxicity. However, there are conflicting data concerning the renal effects of SFZ which might be a dose dependent effect. In this context, 400 and 600 mg/kg/day SFZ induced nephrotoxicity as evidenced by Niknahad et, al (Niknahad et al. 2017). However, Demirbilek, et al. and Camara, et al. demonstrated renoprotective effects of 100 mg/kg/day SFZ (Cámara-Lemarroy et al. 2009; Demirbilek et al. 2007).

In summary, many evidences have proven the potential role of NF-κB in cisplatin-induced nephrotoxicity (Altındağ 2022; Zhao and Wen 2018). In which, NF-κB acts as a central link between inflammation, fibrosis and apoptosis by inducing the pro-inflammatory cytokine TNF-α that promotes the expression of further inflammatory cytokines like IL-6. Subsequently, TNF-α up-regulates the fibrotic insult represented as elevation of TGF-β1. Furthermore, Chronic or excessive NF-κB activation with its subsequent induction of miRNA-375 and repression of HNF-1B, in addition to sustained inflammation, fibrosis and oxidative stress promotes renal cells’ apoptosis expressed as elevation of caspase-3 level, creating a viscous cycle that drives progression of renal injury. Thus, the present study aimed to investigate the renal effects of NF-κB inhibition using sulfasalazine (Zhang et al. 2023). Although sulfasalazine is well-known for nephrotoxicity at dose of 400 and 600 mg/kg (Niknahad et al. 2017), its renal effect at dose of 100 mg/kg remains obscure.

Based on these considerations, the present study aimed to evaluate the safety of sulfasalazine (SFZ) at a dose of 100 mg/kg with respect to renal function. Additionally, it sought to determine whether SFZ (100mg/kg) treatment could mitigate cisplatin-induced neohrotoxicity characterized by NF-κB-mediated inflammatory, fibrotic, and apoptotic processes. Furthermore, the present study investigated the underlying signaling pathways and molecular mechanisms through which SFZ may exert its inhibitory effect on NF-κB activation.

## Materials and methods

### Animals

For this study, forty-eight adult male Sprague–Dawley rats weighing between 140 and 200 g were used. The animals were obtained and the procedures were carried out at animal house of Medical Research Institute-Alexandria University (Alexandria, Egypt). The experiment was performed after approval of institutional Ethics Research Committee of the Faculty of Pharmacy, Damanhur University (Damanhur, Egypt) (Ref. No. 223PO33). Rats were housed in a 12-h light/dark cycle with standard environmental conditions including daylight, temperature and humidity for two weeks before the experiment to allow for acclimatization and to guarantee typical behavior and growth. Rat chow and tap water were freely available to the animals. The food for animals was a typical chow with well-balanced ingredients. There are no clinical studies or patient data in the manuscript.

### Drugs and chemicals

Sulfasalazine (Colosalazine-EC®, El kahira Pharmaceutical company), Cisplatin (Cis) (Cisplatine® Mylan 10 ml vial, Oncotec Pharma production, Germany), Thiopental sodium (Thiopental Sodium®, Sandoz Pharmaceuticals, Basel, Switzerland). A 100 mg/kg working solution was freshly prepared by triturating Colosalazine ® tablets in sterile physiological saline.. Other chemicals and reagents used in biochemical tests were of analytical grade. Details of quantitative analysis kits are under biochemical analysis section.

### Experimental design

Animals received an intraperitoneal injection of cisplatin at a dose of 6 mg/kg, which is well documented to induce nephrotoxicity in rats (Hosseinian et al. 2016; Mohan et al. 2006; Moneim et al. 2019). Sulfasalazine was suspended in sterile physiological saline and administered to animals by oral gavage at the doses of 100 mg/kg/day. The dose of Sulfasalazine used in this study was selected on the basis of the previous studies (Cámara-Lemarroy et al. 2009; Demirbilek et al. 2007). Animals were randomly divided into four groups (n = 12): (i) Negative control (received an oral daily dose of normal saline for 19 days., n = 12), (ii) Cisplatin-treated group (received a single dose of cisplatin 6 mg/kg, i.p. at the 6th day of experiment., n = 12) (Moneim et al. 2019), (iii) Sulfasalazine only-treated group (received SFZ only as an oral daily dose of 100 mg/kg, treatment lasts for 19 days., n = 12) and (iv) Cis + SFZ (given a single dose of cisplatin 6 mg/kg, i.p. that was preceded with an oral daily dose of sulfasalazine (100 mg/kg/day) for 5 days and followed by the same dose of sulfasalazine for another 14 days afterwards., n = 12). In each group, half of the rats were sacrificed 96 h after cisplatin administration (which represents the maximal peak insult of cisplatin-induced nephrotoxicity) and the other half were sacrificed 14 days after cisplatin administration (which represents the long-lasting insult of cisplatin-induced nephrotoxicity and for tracing potential adaptive fibrotic changes of cisplatin) (El Hilaly et al. 2004; Moneim et al. 2019; Parasuraman 2011).

### Sample preparation

#### Blood and tissue preparation

**Fig. 1:**
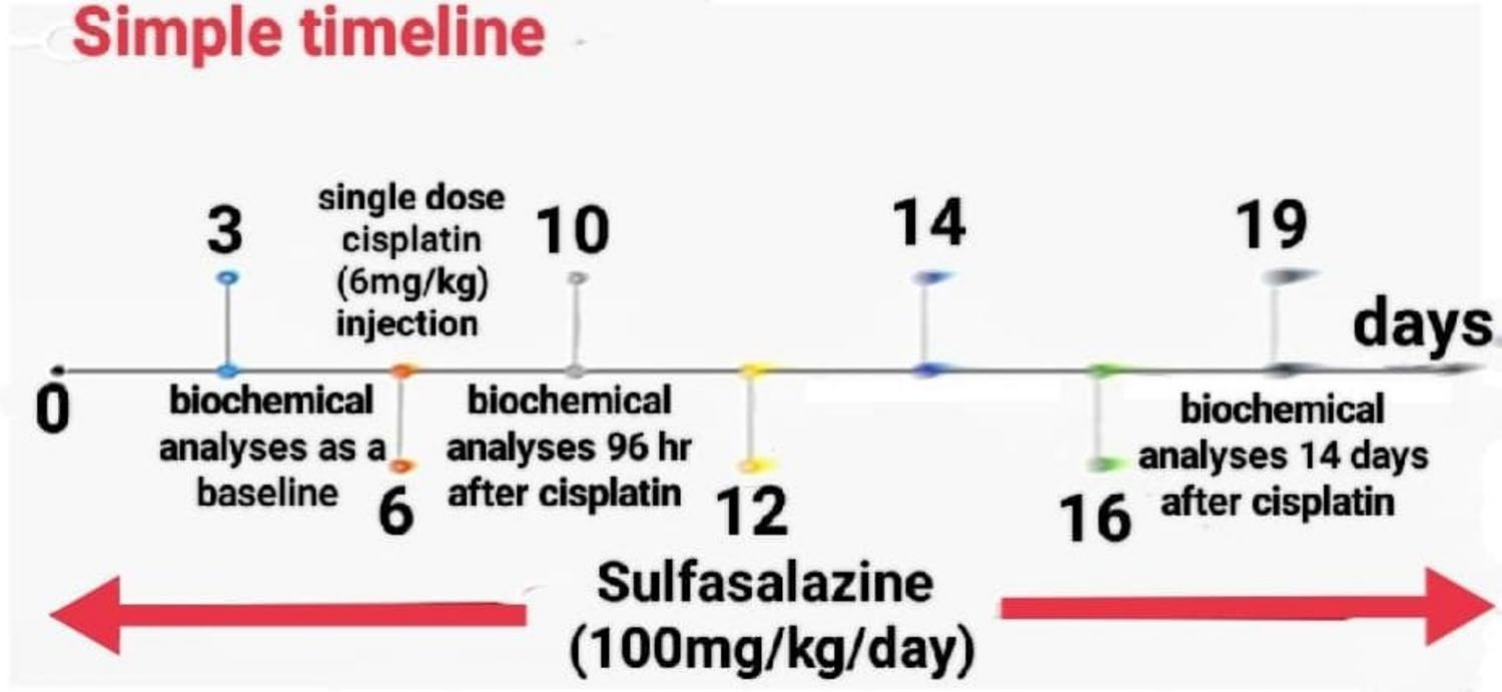
Schematic diagram for dosing and timing

Blood samples were drawn from all rats to be biochemically evaluated at 3 time-points; before cisplatin injection as a baseline, 96 h and 14 days after cisplatin injection, as shown in schematic diagram (Fig. 1). The serum urea and creatinine levels were calculated using the delta change rather than the absolute value at each time interval. Under mild anesthesia with thiopental sodium (50 mg/kg, i.p.) (Helmy et al. 2014b), blood samples were obtained from the orbital plexus of the animals. At the end of experiment for each time interval, rats were euthanized with an over dose of the anesthetic drug (thiopental 150 mg/kg, i.p.) (de Brito et al. 2020). Non-heparinized capillary tubes were used to collect blood samples. The recovered serum was kept at −70 °C until the urea and creatinine levels were analyzed. Following euthanasia and collection of blood samples, The animals were dissected and the two kidneys were promptly removed. The left kidney was divided longitudinally into two halves. One of the two longitudinally divided sections of the left kidney was preserved in a 10% formaldehyde solution in saline and then embedded in paraffin blocks within 24 h for histopathological analysis. The other half together with the right kidney were washed with ice-cold saline, blotted dry, weighed and homogenized (10%) with ice-cold saline to create 40% kidney homogenate. Until molecular analysis, kidney homogenates were divided into portions and immediately stored at −80 C.

#### Histopathological tissue preparation

For light microscopic evaluation, one half of the longitudinal sections of the left kidneys was preserved in 10% formaldehyde saline and then dehydrated and embedded in paraffin wax blocks within 24 h. 5 mm-thick kidney sections were cut and stained with hematoxylin–eosin (H&E) in order to identify any histopathological alterations in renal tissues (Bancroft 2013; Helmy et al. 2014b; Moneim et al. 2019; Suvarna et al. 2018).

### Sample analysis

#### Biochemical analysis

To evaluate kidney function, serum creatinine and urea levels were analyzed using BioSystems® assay kits (Biosystems S.A., Barcelona, Spain).

#### Molecular analysis

##### Enzyme-linked immunosorbent assay (ELISA)

Linked Immunosorbent Assay (ELISA) kits were used for determination of renal levels of Toll-like receptor 4 (TLR-4; CUSABIO; cat #: CSB-E15822r), Nuclear Factor Kappa B p65 (NF-kB p65; Elabscience, USA; cat #: E-EL-R0674), tumour necrosis factor-α (TNF-α; Chongqing Biospes Co., Ltd., China; cat #: BEK1214), interleukin 6 (IL-6; NOVUSBIO, US; cat #: NBP1-92,697), transforming growth factor-beta1 (TGF-B1; CUSABIO; cat #: CSB-E04727r), (caspase-3; CUSABIO; cat #: CSB-E08857r), phosphorylated AMP-activated protein kinase (p-AMPK; LSBio; cat #: LS-F36060), Sirtuin 1 (SIRT-1; Elabscience Biotechnology Inc., USA; cat #: E-EL-R1102), hepatocyte nuclear factor-1β (HNF-1; Southern California, San Diego (USA) cat #: MBS459885) and Total Anti-oxidant Capacity (TAC) as instructed by the manufacturer.

##### Reverse transcription and quantitative polymerase chain reaction (RT-qPCR)

Sequencing and quantitative real time PCR were used for determination of renal levels of microRNA 132-3p (Qiagen, Germany; cat #: YP00206035) and microRNA 375 (Qiagen, Germany; cat #: YP00204362). The housekeeping gene is U6 snRNA (reference gene). U6 is one such reference gene that is often used.

#### Histopathological Examination

Haematoxylin and eosin (H&E)-stained sections were examined under a light microscope for histopathological alterations, and the kidney’s histological damage was blindly evaluated in a semi-quantitative manner. Six randomly selected sections from each kidney were examined at 400 × magnification. Each section was scored on a scale of 0 to 3 for tubular cell necrosis, inflammatory cell infiltration, and atrophic features of glomerular injury. The mean of the six scores was calculated for each kidney. Each histopathological change was scored as (0) absent, (1) mild, (2) moderate, or (3) severe. For these three criteria, the total histological score varied from 0 (normal) to 9 (severely damaged). (Bancroft 2013; Helmy et al. 2015; Moneim et al. 2019; Suvarna et al. 2018).

### Statistical analysis

All of the result data are expressed as mean ± SD of 6 rats in each time interval. Regarding functional and molecular parameters, the significant difference between groups was analyzed quantitatively using One-Way Analysis of Variance (ANOVA) followed by Tukey's test for parameters measured at one time interval (96 h) but using Two-Way Analysis of Variance (ANOVA) followed by Tukey's test for parameters measured at both time intervals (96 h and 14 days). Concerning the histopathological scoring, the significant difference was analyzed semi-quantitatively using Mann–whitney U test between different treatment groups at the same time interval but using Wilcoxon signed-rank test for the same treatment group between the two time intervals. Differences with values of p < 0.05 were considered statistically significant. Calculations were done using a computer software program Graph PAD Prism (Version 8).

## Results

### Biochemical changes of kidney functions parameters

**Fig. 2:**
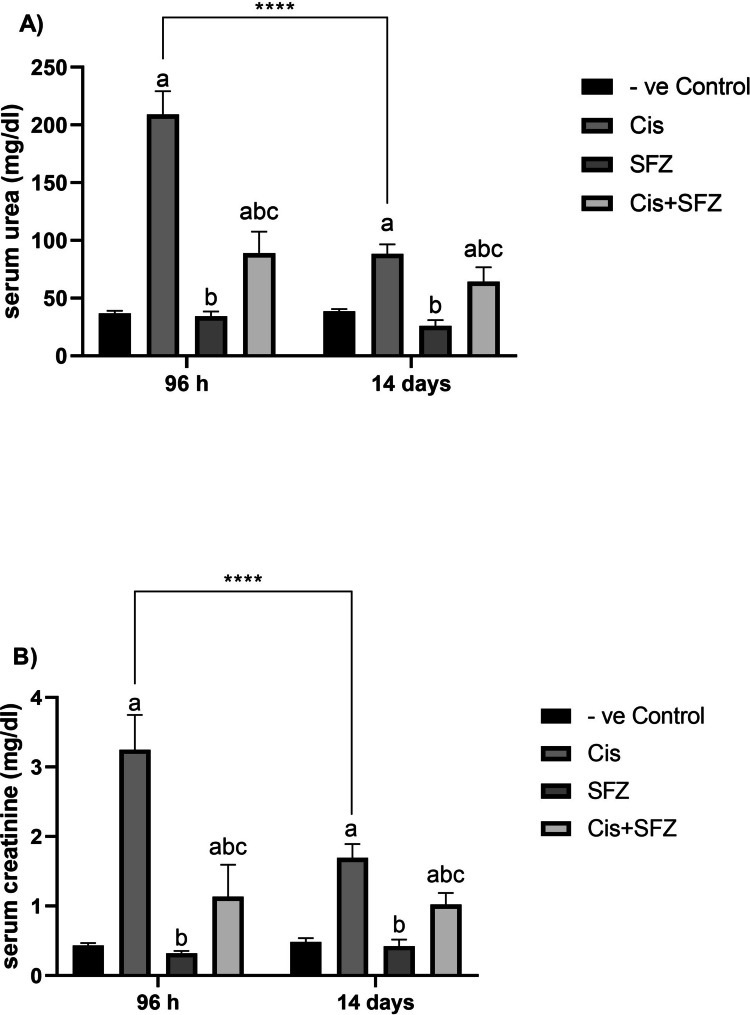
Effects of different treatment regimens on serum urea (mg/dl) (**A**) and serum creatinine (mg/dl) (**B**) obtained from male rats at 96 h and 14 d following the administration of saline (control), cisplatin (6 mg/kg, i.p.), sulfasalazine (100 mg/kg by oral gavage) and cisplatin + sulfasalazine (Cisplatin, 6 mg/kg, single dose at the 6th day of the experiment and SFZ, 100 mg/kg, starting 5 days before and continued for 4 or 14 days after the cisplatin dose). Values are means of 6 observations/each time interval expressed as mean ± SD (n = 6). Statistical analysis was carried out using two-way ANOVA followed by Tukey’s multiple comparisons test at (p < 0.05). a, b, and c denote significant difference vs. control, cisplatin and sulfasalazine values, respectively. * denotes significant difference between the same treatment on different time intervals

The significant difference was investigated regarding serum urea and creatinine levels between different treatment groups at the same time interval and the same treatment on both time intervals. At 96 h following cisplatin administration, there were a significant increase in serum urea and creatinine concentrations in cisplatin-treated rats (6 mg/kg, i.p.) which were (209.33 ± 20 mg/dl (p < 0.0001) for urea and 3.24 ± 0.49 mg/dl (p < 0.0001) for creatinine) compared to (36.7 ± 2.42 mg/dl and 0.43 ± 0.036 mg/dl, respectively) in the negative control (saline-treated) group. Co-administration of sulfasalazine (100 mg/kg by oral gavage) with cisplatin (6 mg/kg, i.p.) resulted in a significant reduction of serum urea and creatinine levels which were (89.1 ± 18.5 mg/dl (p < 0.0001) for urea and 1.13 ± 0.46 mg/dl (p < 0.0001) for creatinine) compared to (209.33 ± 20 mg/dl and 3.24 ± 0.49 mg/dl, respectively) in rats treated with cisplatin alone. On the other hand, after 14 days following cisplatin injection, cisplatin treatment (6 mg/kg, i.p.) led to a significant increase in serum urea and creatinine levels which were (88.5 ± 7.98 mg/dl (p = 0.0002) for urea and 1.69 ± 0.19 mg/dl (p < 0.0001) for creatinine) compared to (38.6 ± 1.7 mg/dl and 0.48 ± 0.05 mg/dl, respectively) in the negative control group. Compared to rats treated with cisplatin alone, co-administration of sulfasalazine with cisplatin succeeded to significantly decrease serum urea and creatinine levels which were (64.3 ± 12.3 mg/dl (p = 0.019) for urea and 1.02 ± 0.16 mg/dl (p = 0.0015) for creatinine) compared to (88.5 ± 7.98 mg/dl and 1.69 ± 0.19 mg/dl, respectively) in the cisplatin group. On both time intervals, safety of sulfasalazine at dose of 100 mg/kg on renal functions was demonstrated by the non-significant differences of serum urea and creatinine levels compared to the negative control (saline-treated) group of rats. Cisplatin-only treated group showed a significant difference in serum urea and creatinine levels between different time intervals (at 96 h and 14 days of its administration) by (0.58-fold decrease for urea and 0.48-fold decrease for creatinine) (Fig. 2A-B).

### Changes in the inflammatory markers TLR-4, NF-κB, TNF-α and IL-6 levels

**Fig. 3:**
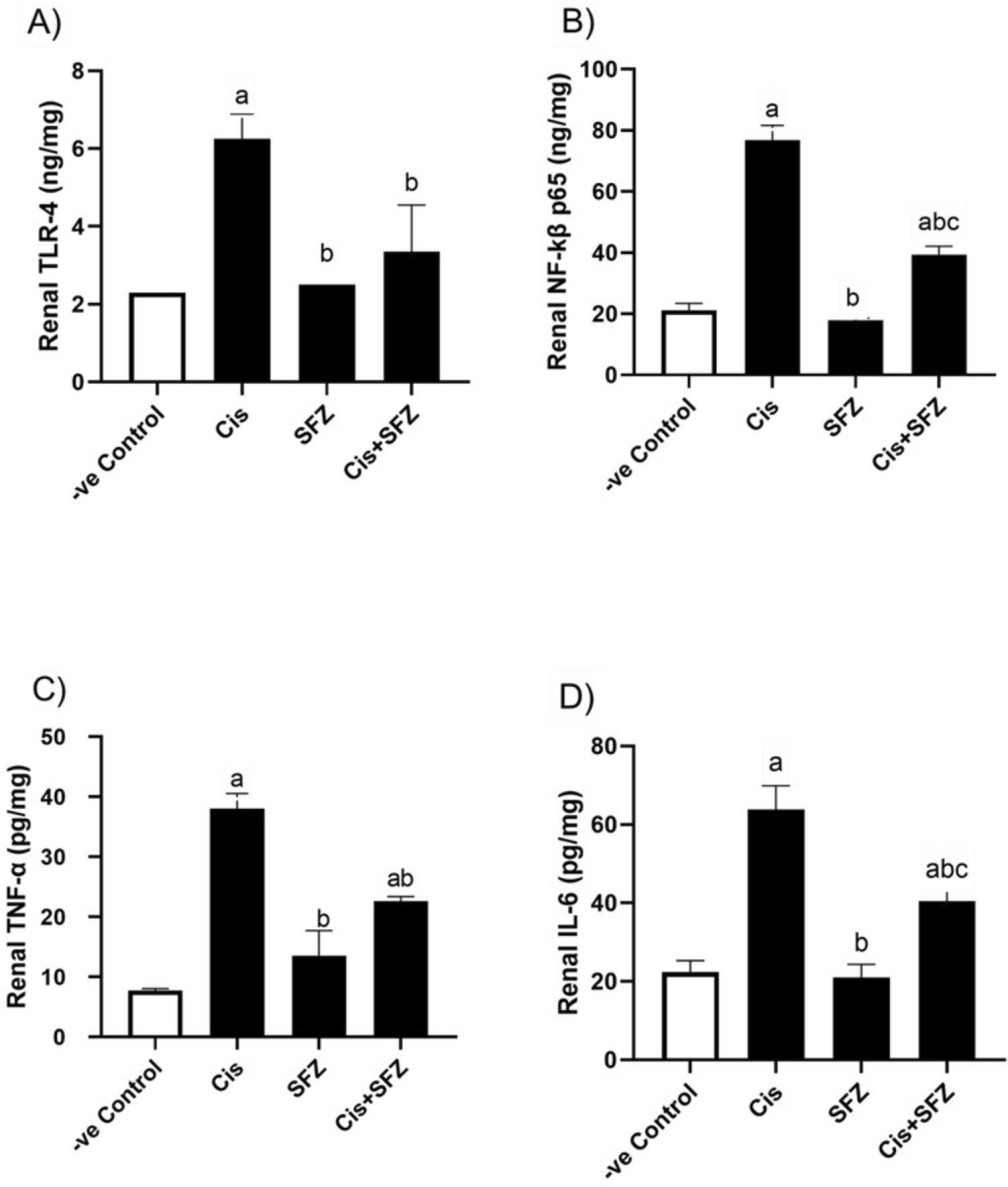
Effects of different treatment regimens on Renal Toll like receptor-4 concentration (ng/mg) (**A**), Renal Nuclear factor-kappa β concentration (ng/mg) (**B**), Renal tumor necrosis factor alpha concentration (pg/mg) (**C**) and Renal interleukin-6 concentration (pg/mg) (**D**) in kidney homogenates obtained from male rats at 96 h following the administration of saline (control), cisplatin (6 mg/kg, i.p), sulfasalazine (100 mg/kg, PO), and Cisplatin + sulfasalazine. Values are means of 6 observations expressed as mean ± SD (n = 6). Statistical analysis was carried out using one-way ANOVA followed by Tukey's multiple comparisons test at (p < 0.05). a, b, and c denote significant difference vs. control, cisplatin and sulfasalazine values, respectively

To further explore the nephroprotective effects of sulfasalazine on molecular mechanisms underlying cisplatin-induced nephrotoxicity, the renal protein expression of TLR-4, NF-κB, TNF-α and IL-6 was investigated, as shown in Fig. 3. The resulting analysis showed that, cisplatin induced a significant increase in protein expression levels which were (6.25 ± 0.63 ng/mg for TLR4, 76.85 ± 4.74 ng/mg for NF-κB, 38 ± 2.5 pg/mg for TNF-α and 63.82 ± 6 pg/mg for IL-6) compared to (2.25 ± 0.07 ng/mg, 21.2 ± 2.26 ng/mg, 7.74 ± 0.32 pg/mg and 22.35 ± 2.97 pg/mg, respectively) in negative control group (p = 0.0142, 0.0002, 0.0009 and 0.0017 for TLR-4, NF-κB, TNF-α and IL-6, respectively). On the other hand, sulfasalazine at dose of 100 mg/kg showed a non-significant difference compared to normal saline treated group. Co-administration of sulfasalazine (100 mg/kg by oral gavage) with cisplatin (6 mg/kg, i.p.) succeeded to significantly decrease protein expression levels which were (3.35 ± 1.2 ng/mg for TLR-4, 39.4 ± 2.68 ng/mg for NF-κB, 22.65 ± 0.7 pg/mg for TNF-α and 40.52 ± 2.5 pg/mg for IL-6) compared to (6.25 ± 0.63 ng/mg, 76.85 ± 4.74 ng/mg, 38 ± 2.5 pg/mg and 63.82 ± 6 pg/mg, respectively) in the cisplatin group (p = 0.0429, 0.0008, 0.0119 and 0.0144 for TLR-4, NF-κB, TNF-α and IL-6, respectively) (Fig. 3A–D).

### Changes in miRNA-375 and miRNA132-3p

**Fig. 4:**
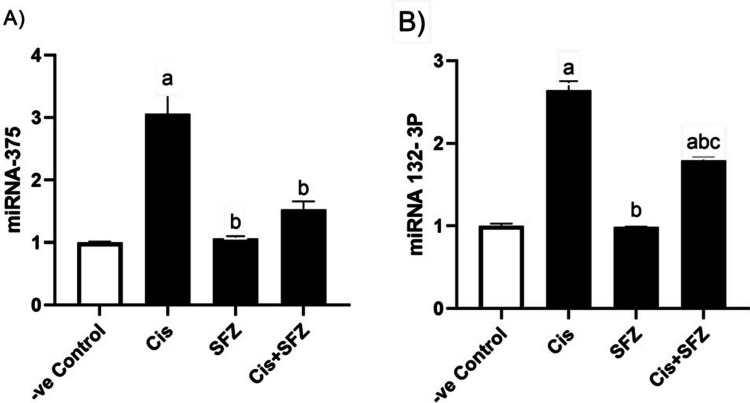
Effects of different treatment regimens on qRT-PCR analysis of renal miR-375 (**A**) and miR-132-3p (**B**) in male rats at 96 h following the administration of saline (control), cisplatin (6 mg/kg, i.p.), sulfasalazine (100 mg/kg, PO), and Cisplatin + sulfasalazine. Values are means of 6 observations expressed as mean ± SD (n = 6). Statistical analysis was carried out using one-way ANOVA followed by Tukey’s multiple comparisons test. a, b, and c denote significant difference (P < 0.05) vs. control, cisplatin and sulfasalazine values, respectively

The renal miR-375 and miR-132-3p genes were investigated using qRT-PCR analysis as shown in Fig. 4. At 96 h following cisplatin administration, cisplatin treatment (6 mg/kg, i.p.) led to a significant increase of miR-375 and miR-132-3p which were (3.06 ± 0.29 and 2.64 ± 0.11, respectively) compared to (1 ± 0.014 and 1 ± 0.028, respectively) in negative control group (p = 0.0008 and 0.0001 for miR-375 and miR-132-3p, respectively). Sulfasalazine at dose of 100 mg/kg showed a non-significant difference compared to normal saline treated group regarding renal miR-375 and miR-132-3p genes. Compared to rats treated with cisplatin alone, co-administration of sulfasalazine with cisplatin succeeded to significantly decrease miR-375 and miR-132-3p which were (1.53 ± 0.127 and 1.795 ± 0.035, respectively) compared to (3.06 ± 0.29 and 2.64 ± 0.11, respectively) in cisplatin group (p = 0.0025 and 0.0005 for miR-375 and miR-132-3p, respectively) (Fig. 4A–B).

### Changes in HNF-1B, SIRT1, p-AMPK and TAC protein level

**Fig. 5:**
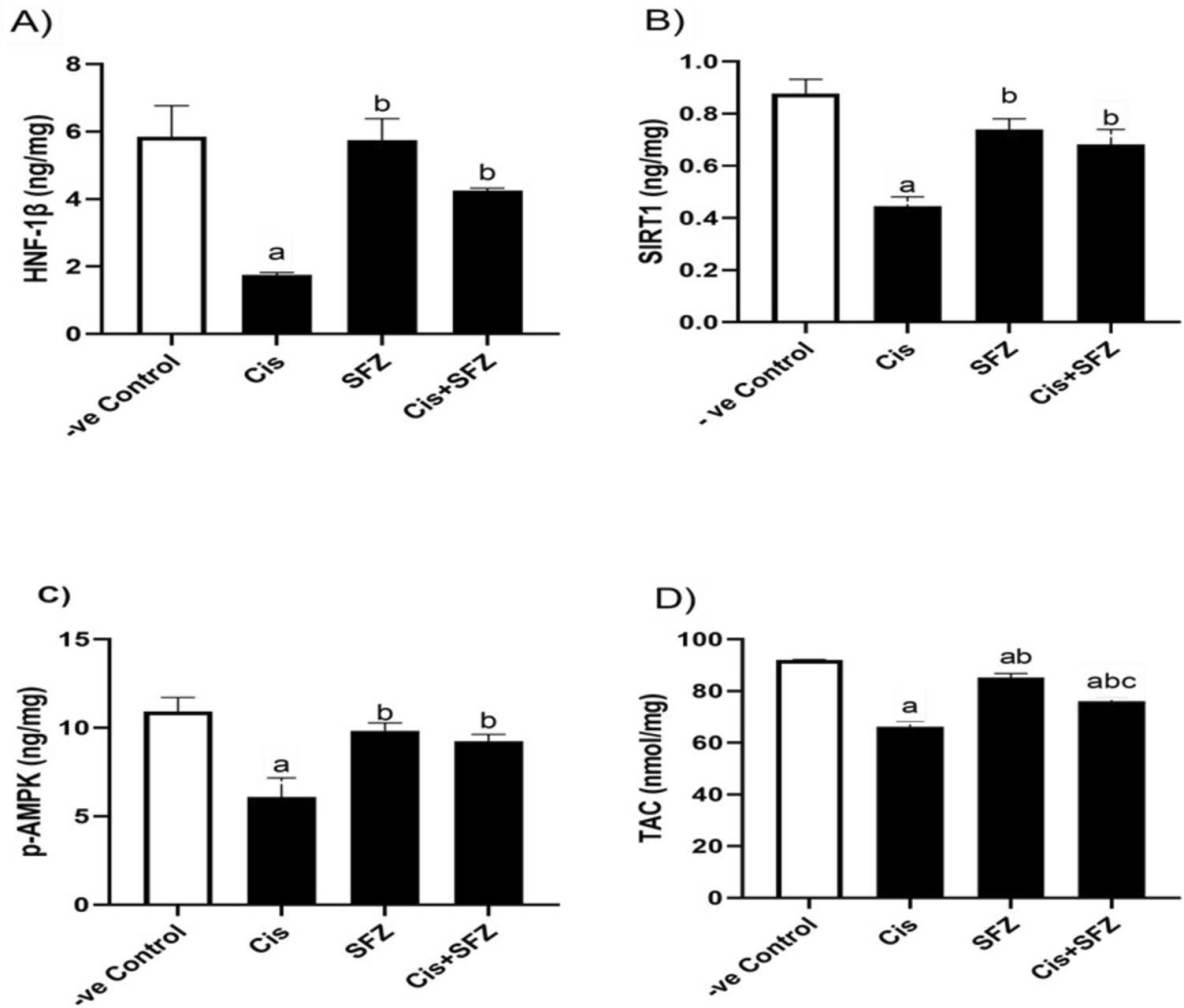
Effects of different treatment regimens on renal hepatocyte nuclear factor-1 beta (HNF-1B) (ng/mg) (**A**), sirtuin-1 (SIRT1) (ng/mg) (**B**), cAMP-activated protein kinase (AMPK) (ng/mg) (**C**) and total anti-oxidant capacity (TAC) (nmol/mg) (**D**) in kidney homogenates obtained from male rats at 96 h following the administration of saline (control), cisplatin (6 mg/kg, i.p.), sulfasalazine (100 mg/kg, PO) and Cisplatin + sulfasalazine. Values are means of 6 observations expressed as mean ± SD (n = 6). Statistical analysis was carried out using one-way ANOVA followed by Tukey's multiple comparisons test at (p < 0.05). a, b, and c denote significant difference (P < 0.05) vs. control, cisplatin and sulfasalazine values, respectively

The renal protein levels of HNF-1B, SIRT1, p-AMPK and total anti-oxidant capacity were investigated using ELISA analysis as shown in Fig. 5. After 96 h following cisplatin administration, cisplatin treatment (6 mg/kg, i.p.) significantly repressed HNF-1B, SIRT1, p-AMPK and TAC expression levels which were (1.75 ± 0.07 ng/mg, 0.4455 ± 0.04 ng/mg, 6.095 ± 1.06 ng/mg and 66.26 ± 1.75 nmol/mg, respectively) compared to (5.85 ± 0.92 ng/mg, 0.877 ± 0.05 ng/mg, 10.925 ± 0.8 ng/mg and 85.5 ± 1.9 nmol/mg, respectively) in negative control (saline-treated) group (p = 0.0065, 0.003, 0.0093 and 0.0039 for HNF-1B, SIRT1, p-AMPK and TAC, respectively). In sulfasalazine (100 mg/kg) only-treated group, sulfasalazine showed a non-significant difference compared to normal saline treated group regarding renal HNF-1B, SIRT1, p-AMPK and TAC protein level. Compared to rats treated with cisplatin alone, co-administration of sulfasalazine with cisplatin succeeded to significantly increase HNF-1B, SIRT1, p-AMPK and TAC expression which were (4.25 ± 0.07 ng/mg, 0.6825 ± 0.06 ng/mg, 9.245 ± 0.38 ng/mg and 76.12 ± 1.29 nmol/mg, respectively) compared to (1.75 ± 0.07 ng/mg, 0.4455 ± 0.04 ng/mg, 6.095 ± 1.06 ng/mg and 66.26 ± 1.75 nmol/mg, respectively) in cisplatin group (p = 0.0373, 0.0268, 0.0415 and 0.0422 for HNF-1B, SIRT1, p-AMPK and TAC, respectively) (Fig. 5A-D).

### Changes in the apoptotic marker caspase-3 protein content

**Fig. 6:**
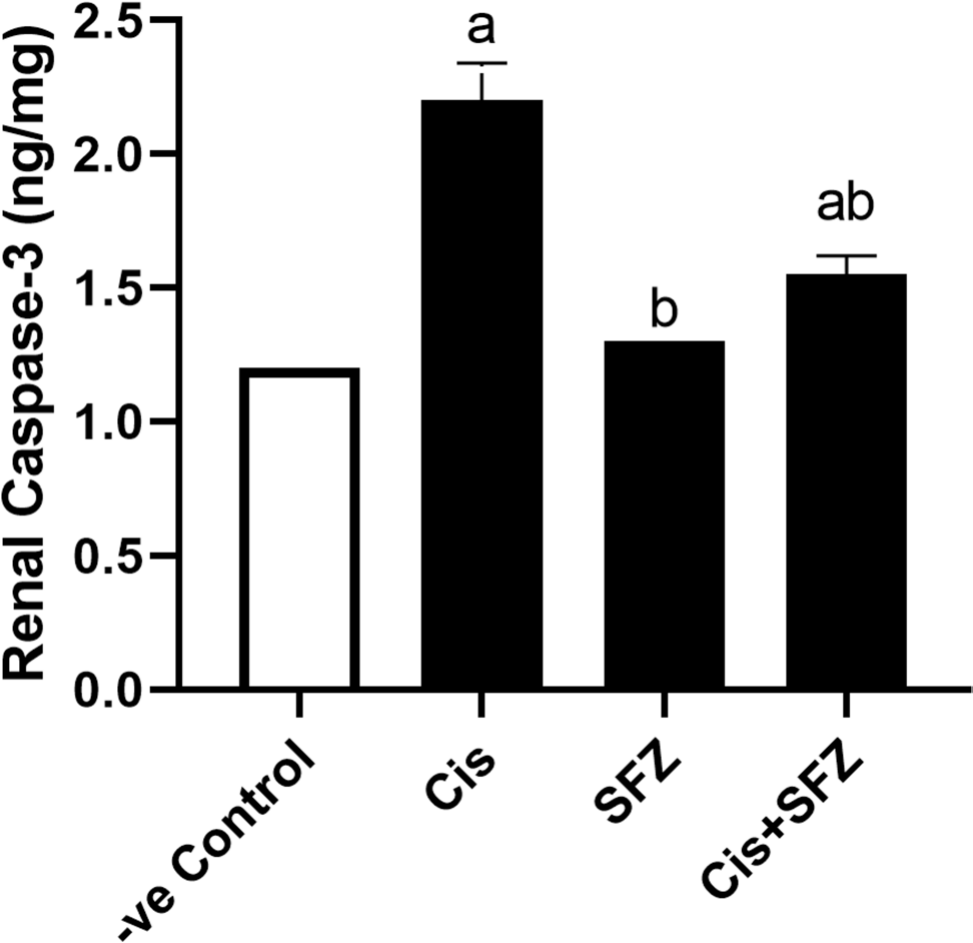
Effects of different treatment regimens on the renal protein level of caspase-3 (ng/mg) in kidney homogenates obtained from male rats at 96 h following the administration of saline (control), cisplatin (6 mg/kg, i.p.), sulfasalazine (100 mg/kg, PO), and Cisplatin + sulfasalazine. Values are means of 6 observations expressed as mean ± SD (n = 6). Statistical analysis was carried out using one-way ANOVA followed by Tukey’s multiple comparisons test at (p < 0.05). a, b and c denote significant difference (P < 0.05) vs. control, cisplatin and sulfasalazine values, respectively

The renal protein level of the apoptotic marker caspase-3 was investigated using ELISA analysis as shown in Fig. 6. At 96 h following cisplatin administration, cisplatin treatment (6 mg/kg, i.p.) induced a significant increase in caspase-3 level which was (2.2 ± 0.14 ng/mg with p = 0.0008) compared to (1.2 ± 0.007 ng/mg) in negative control group. Safety of sulfasalazine at dose of 100 mg/kg on renal functions was demonstrated by the non-significant differences of renal caspase-3 level compared to normal saline-treated group. Compared to rats treated with cisplatin alone, co-administration of sulfasalazine with cisplatin succeeded to significantly decrease caspase-3 level which was (1.55 ± 0.07 ng/mg with p = 0.004) compared to (2.2 ± 0.14 ng/mg) in cisplatin group (Fig. 6).

### Changes in the fibrotic marker TGF-β1 level

**Fig. 7:**
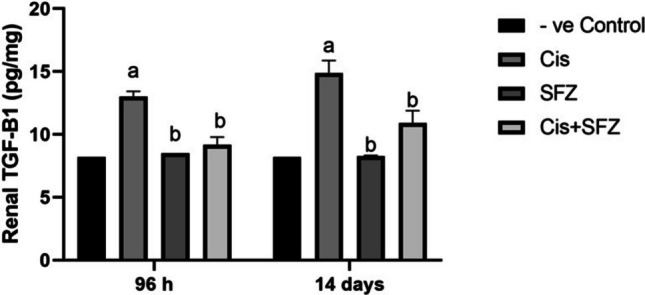
Effects of different treatment regimens on transforming growth factor-beta1 (pg/mg) in kidney homogenates obtained from male rats at 96 h and 14 days following the administration of saline (control), cisplatin (6 mg/kg, i.p.), sulfasalazine (100 mg/kg, PO), and Cisplatin + sulfasalazine. Values are means of 6 observations in each time interval, expressed as mean ± SD (n = 6). Statistical analysis was carried out using two-way ANOVA followed by Tukey's multiple comparisons test at (p < 0.05). a, b and c denote significant difference (P < 0.05) vs. control, cisplatin and sulfasalazine values, respectively

The significant difference regarding renal protein level of TGF-β1 was investigated between different treatment groups at the same time interval and the same treatment on both time intervals using ELISA analysis as shown in Fig. 7. Compared to control (saline-treated) group, there were a significant increase in the renal levels of TGF-β1 in cisplatin-treated rats which were 13 ± 0.42 pg/mg (p = 0.0008) and 14.89 ± 0.98 pg/mg (p = 0.0023) at 96 h and 14 days of cisplatin injection, respectively compared to (8.205 ± 0.007 pg/mg and 8.21 ± 0.014 pg/mg, respectively) in the negative control group. Compared to the negative control (saline-treated)group, sulfasalazine did not show significant difference in renal TGF-β1 level. Compared to rats treated with cisplatin alone, co-administration of sulfasalazine with cisplatin succeeded to significantly decrease the renal level of TGF-β1 which were 9.15 ± 0.63 pg/mg (p = 0.0019) and 10.91 ± 0.98 pg/mg (p = 0.0159) at 96 h and 14 days, respectively compared to (13 ± 0.42 pg/mg and 14.89 ± 0.98 pg/mg, respectively) in the cisplatin group (Fig. 7).

### Renal histopathological changes

**Fig. 8:**
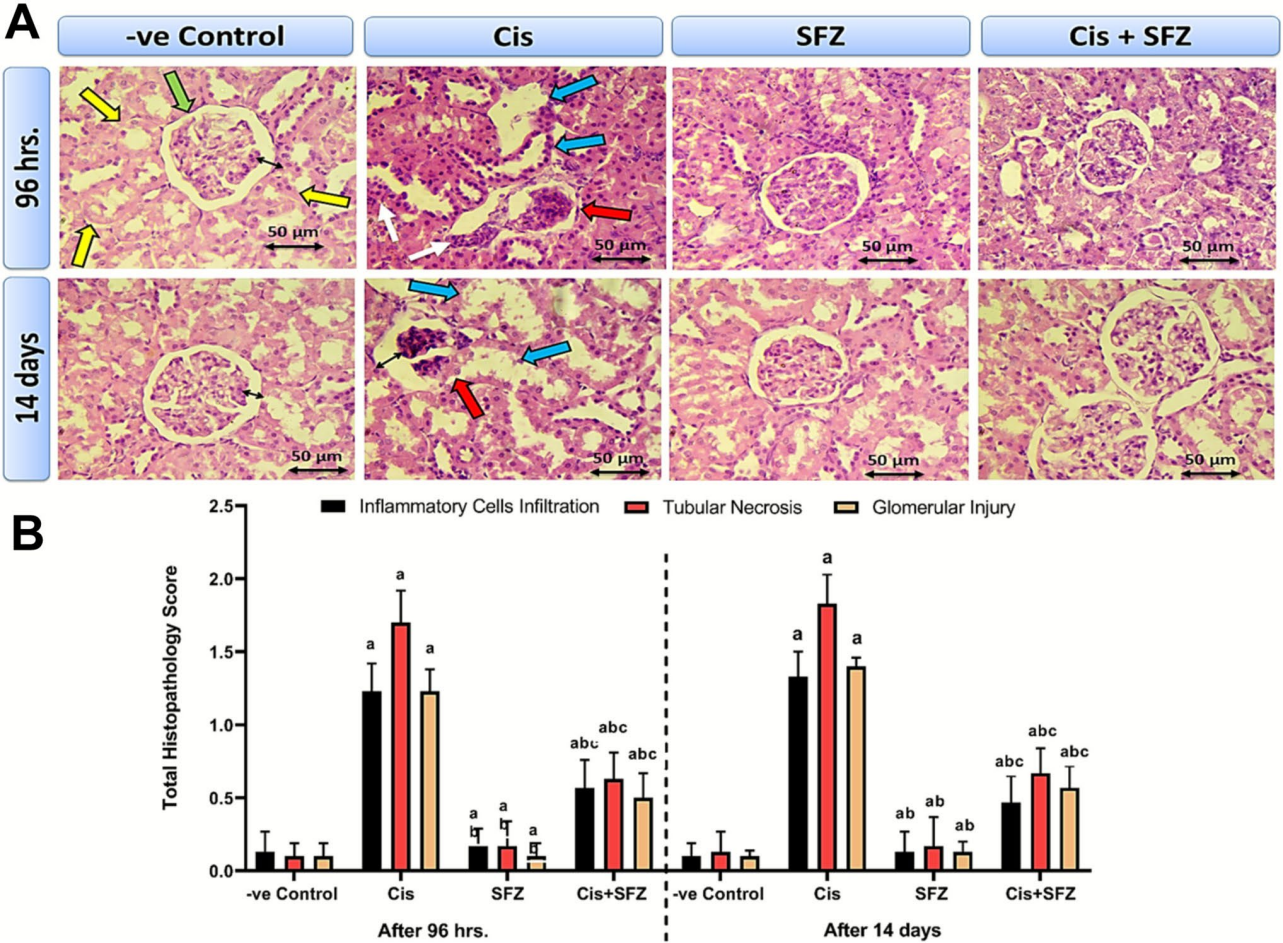
(**A**) Photomicrographs (100x) of renal histopathological changes (hematoxylin–eosin) associated with different treated groups. Negative Control group showing normal renal tissues including normal renal tubules (yellow arrows) and normal glomeruli (green arrow) with homogenous mesangial matrix and preserved Bowman’s space (↔). Cisplatin-treated group exhibited mild interstitial nephritis represented by few fibroblasts and mononuclear cell infiltrations (white arrows), desquamation of the brush border of tubules forming cast (black arrows), moderate vacuolation of the distal convoluted renal tubules (light blue arrows) and Cystic dilatation of the Bowman capsule (red arrows) with obliteration of Bowman’s space. The histological changes of cisplatin were alleviated by pre-treatment with SFZ. (**B**) The total histopathological score which scored as (0) absent, (1) mild, (2) moderate, or (3) severe. Statistical analysis was carried out semi-quantitatively using Mann–whitney U test between different treatment groups at the same time interval while using Wilcoxon signed-rank test for the same treatment group between both time intervals. The number of replicates per group (n = 6/each time interval)

Concerning the histopathological changes, Fig. 8 shows (A) the histopathological potomicrographs at 96 h and 14 days after cisplatin administration and (B) the total histopathological score at 96 h and on day 14. At both time intervals, the cisplatin group’s histopathological damage scores for inflammatory cell infiltration, acute tubular necrosis, and glomerular injury were significantly higher than those of the negative control group. In contrast, safety of sulfasalazine at dose of 100 mg/kg on renal functions was demonstrated by the non-significant differences of renal histological study compared to the negative control group. Consequently, pretreatment with sulfasalazine significantly reduced the histopathological damage score of cisplatin at both time intervals (Fig. 8A-B).

## Discussion

One of the pathophysiological bases of cisplatin-induced nephrotoxicity is the activation of the transcription factor nuclear factor-kappa B (NF-κB) (Altındağ 2022; Zhao and Wen 2018). In the same context, it has been shown that sulfasalazine is a potent NF-κB inhibitor (Zhang et al. 2023). Based on this notion, this study aimed to evaluate both safety and potential anti-inflammatory and nephroprotective effects of sulfasalazine against cisplatin-induced nephrotoxicity.

The present study proved the safety of sulfasalazine at the dose of 100 mg/kg on renal functions as demonstrated by the non-significant differences of SFZ group compared to the negative control group of rats regarding functional, molecular and structural renal effects. In addition, the current study is the first to reveal that sulfasalazine could significantly alleviate cisplatin-induced nephrotoxicity. There are few reports on sulfasalazine nephroprotection against other nephrotoxic settings like renal ischemia/reperfusion injury and obstructive renal injury characterized by NF-κB-mediated inflammatory and fibrotic processes in rats (Cámara-Lemarroy et al. 2009; Demirbilek et al. 2007).

In context with cisplatin-induced nephrotoxicity, results of the current study demonstrated that cisplatin injection (6 mg/kg) to male rats led to histological changes of the renal cells expressed as glomerular atrophic injury, acute tubular necrosis and inflammatory cellular infiltration of macrophages in kidney tissues. These alterations resulted in a deterioration in kidney functions parameters represented as elevated serum urea and creatinine levels. These findings are in parallel to those reported by Helmy. et al. and Moneim. et al., indicating cisplatin-induced nephrotoxicity (Helmy et al. 2014a; Helmy et al. 2014b; Moneim et al. 2019). In the same context, safety of sulfasalazine at dose of 100 mg/kg on renal functions was demonstrated by the non-significant differences of renal function parameters and renal histological study compared to the negative control group. In addition, the current study is the first to reveal that sulfasalazine could significantly alleviate cisplatin-induced nephrotoxicity manifested as a significant decrease in serum urea and creatinine compared to cisplatin-only treated group. This was supported by the histological sections showing normal morphology. These histopathological changes were scored as (0) absent, (1) mild, (2) moderate, or (3) severe (Bancroft 2013; Suvarna et al. 2018). So, The present study suggested that sulfasalazine can be used as a nephroprotective agent by improving these structural changes induced by cisplatin.

Concerning the time factor, the current study is the first to highlight that, the nephrotoxic effect of cisplatin was more pronounced in the maximal peak insult at 96 h than the long-lasting insult after 14 days. The nephrotoxic effect of cisplatin, as demonstrated by the two surrogate renal functional parameters (serum urea and creatinine levels), was more obvious 96 h after cisplatin than 14 days after cisplatin. However, the maximal peak insult of cisplatin after 96 h showed non-significant differences compared to the long-lasting insult after 14 days regarding the fibrotic marker TGF-β1 level. On the other hand, pretreatment with sulfasalazine lowered the renal function parameters and the fibrotic marker after both time intervals non-significantly. The present findings support using sulfasalazine before, during and after the critical period of cisplatin administration.

Interestingly, the results obtained from this study revealed that SFZ at the dose of 100 mg/kg offered a reno-protective effect against cisplatin-induced nephrotoxicity which could be related to its inhibition of NF-κB-p65. The inhibitory effect of SFZ on NF-κB-p65 can preserve the renal function through downstream attenuation of inflammatory pathway (TLR-4/NF-κB/TNF-α/IL-6 axis), fibrotic pathway (TGF-β1/TNF-α axis) and apoptotic pathway (NF-κB/miRNA-375/HNF-1B/caspase-3 axis) which is not correlated to the time factor.

Concerning the inflammatory pathway, results of the present study demonstrated that acute injury induced by cisplatin injection (6 mg/kg) to male rats significantly increased the renal levels of the inflammatory molecules (TLR-4, NF-κB, TNF-α and IL-6). This result is in line with previous studies which have proved that, both activation of NF-κB-p65 and release of TLR-4 ligand; DAMPs induced by cisplatin could stimulate the transcription of TNF-α with subsequent extensive production of inflammatory cytokines as; IL-6 and thus promotes renal inflammatory injury (Manohar and Leung 2018; Yao et al. 2007). The increased inflammatory response contributes to the pathogenesis and exacerbation of renal tissue damage in cisplatin-administered animals.

The present study revealed that, the concurrent administration of sulfasalazine with cisplatin significantly decreased the renal levels of NF-κB-p65 and its subsequent inflammatory cytokines (TNF-α and IL-6) compared to the cisplatin only-treated group. These findings are in parallel to those reported by Demirbilek. et al. who demonstrated comparable effects of SFZ on obstructive renal injury in rats (Demirbilek et al. 2007). However, the present study, up to our knowledge, is the first to reveal that sulfasalazine could significantly decrease the renal level of TLR-4. So, the present study suggested that sulfasalazine inhibitory effect on cisplatin-induced nephrotoxicity could be at least in part, related to downstream suppression of TLR-4 and NF-κB-p65 through TLR-4/NF-κB/TNF-α/IL-6 axis.

The interplay between NF-κB signaling and oxidative stress is well established, as NF-κB is a redox-sensitive transcription factor activated by reactive oxygen species (ROS), which in turn amplifies inflammatory and apoptotic pathways. Sulfasalazine (SFZ) modulates oxidative stress through dual mechanisms depending on the pathological context. In inflammatory conditions, SFZ exhibits antioxidant properties by reducing lipid peroxidation, malondialdehyde (MDA) and nitric oxide (NO) levels while restoring glutathione (GSH) and superoxide dismutase (SOD), thereby mitigating ROS-driven tissue damage.”(Cheng et al. 2024; Helmy et al. 2025).

Cisplatin-induced upregulated inflammatory pathway extended to upregulate the fibrotic insult in renal tissue as evidenced on molecular level, by elevated TGF-β1 level compared to the negative control group. These findings are in line with those reported by Volarevic. et al. who revealed that, the production of TGF-β1 in cisplatin-injured kidneys was TNF-α-dependent (Volarevic et al. 2019). Moreover, TGF-β1 plays a critical role in renal fibrosis, further promoted TNF-α–driven renal inflammation induced by cisplatin. Similarly, Volarevic. et al. revealed that selective inhibition of TNF-α significantly reduced production of TGF-β1 and attenuated cisplatin-induced nephrotoxicity (Volarevic et al. 2019)..In the same context, the present study demonstrated that sulfasalazine-mediated suppressed inflammatory pathway in Cis + SFZ group extended to down-regulate the fibrotic changes in renal tissue as evidenced on molecular level, by alleviated TGF-β1 level compared to cisplatin only-treated group. These findings are in parallel to those conducted by Demirbilek. et al. who demonstrated comparable effects of SFZ on obstructive renal injury in rats (Demirbilek et al. 2007). Therefore, the molecular results of the current study confirms that SFZ could inhibit TGF-β1–driven renal fibrosis through its downstream inhibition of NF-κB/TNF-α in cisplatin-treated animals.

Concerning the apoptotic pathway, the current results demonstrated that cisplatin injection (6mg/kg) to male rats led to increase the renal levels of NF-κB, miRNA-375 and caspase-3 but reduced the renal levels of hepatocyte nuclear factor 1 homeobox B (HNF-1B) compared to the negative control group. These findings are in line with previous study which have proved that, upon cisplatin exposure, NF-κB induces miRNA-375 expression which in turn represses the nephroprotective gene HNF-1B resulting in increased tubular cell apoptosis represented as a significant higher levels of renal caspase-3 (Hao et al. 2017). Consequently, Hao. et al. demonstrated that the inhibition of NF-κB attenuated cisplatin-induced miRNA-375 expression with subsequent overexpression of the nephroprotective gene HNF-1B which has anti-apoptotic role and reduced the apoptotic marker caspase-3 (Hao et al. 2017). Fortunately, The present study highlighted the potential role of sulfasalazine in attenuating the pro-apoptotic NF-κB/miRNA-375/HNF-1B/caspase-3 pathway through downstream suppression of NF-κB-p65 induced by cisplatin.

Based on the aforementioned findings, it was worthy to further explore the possible underlying signaling pathways and mechanisms that may mediate sulfasalazine inhibition of NF-κB. Interestingly, the present study showed, for the first time, that the modulatory effect of SFZ on NF-κB could be at least in part attributed to its ability to induce upstream modulation of miRNA-132-3p/SIRT-1/NF-κB axis, SIRT1/miR-375/NF-κB axis and p-AMPK/NF-κB axis.

It was reported that Sirtuin-1 (SIRT-1) could act as a negative regulator for NF-κB through its ability to inhibits miRNA-375 (Zhang et al. 2021).

Similarly, Du. et al. and sung. et al. revealed that SIRT-1, in addition to, p-AMPK are negative regulators of NF-κB (Du et al*.*^2019^; Sung et al. 2020). Moreover, Han. et al. demonstrated that cisplatin is a suppressor of SIRT-1 through upregulation of its up-stream regulator miR-132-3p. consequently, cisplatin act as a key inducer of NF-κB (Han et al. 2021). Based on these findings, the present study demonstrated that cisplatin injection (6mg/kg) to male rats significantly increased the renal levels of miRNA-132-3p, miRNA-375 and NF-κB but significantly reduced the renal levels of SIRT-1 and p-AMPK compared to the negative control group. In the same context, the present study demonstrated that the concurrent administration of sulfasalazine with cisplatin significantly increased the renal levels of SIRT-1 and p-AMPK but significantly reduced the renal levels of miRNA-132-3p, miRNA-375 and NF-κB. Interestingly, the present study highlighted the potential role of sulfasalazine in attenuating the upregulated NF-κB induced by cisplatin through down regulating its upstream regulators: miR-132-3p and miRNA-375 while upregulating its upstream regulators: SIRT-1 and p-AMPK. So, the present study suggested that the modulatory/inhibitory effect of SFZ on NF-κB-driven inflammatory, fibrotic and apoptotic renal injury induced by cisplatin could be at least in part attributed to its modulation of p-AMPK/NF-κB axis, miRNA-132-3p/SIRT-1/NF-κB axis and SIRT1/miR-375/NF-κB axis.

## Conclusion

**Fig. 9:**
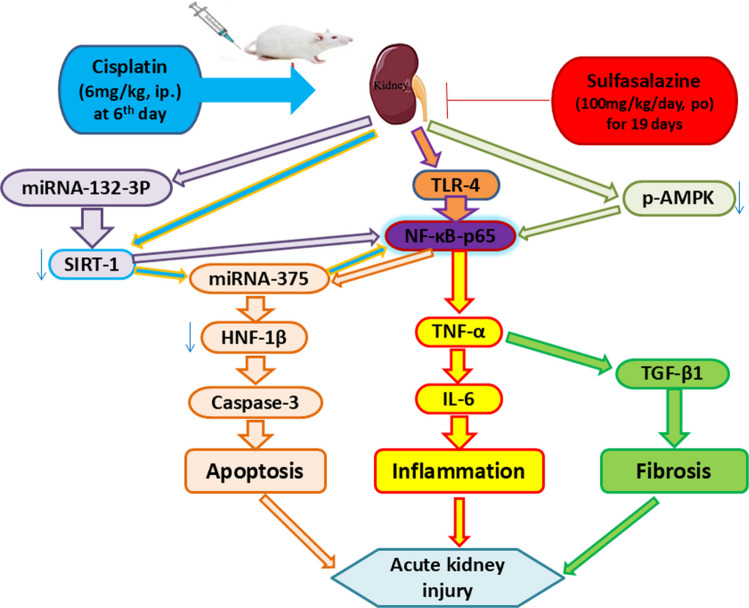
Mechanistic graphical presentation of the reno-protective effects of sulfasalazine (100mg/kg) on cisplatin-induced neohrotoxicity characterized by NF-κB-mediated inflammatory, fibrotic, and apoptotic processes. In addition to summarizing the underlying signaling pathways and molecular mechanisms through which SFZ may exert its inhibitory effect on NF-κB activation

While earlier studies demonstrated the reno-protective effects of Sulfasalazine against renal ischemia/reperfusion injury and obstructive renal injury, the present study is the first to reveal the safety of sulfasalazine at dose of 100 mg/kg on renal functions. In addition, it offers a detailed mechanistic reno-protective effect against cisplatin-induced nephrotoxicity in male rats. This study revealed that the improved functional, biochemical, and structural renal effects rely mainly on sulfasalazine inhibitory effect of pNF-κB-p65 activity. Its attenuation of NF-κB could preserve the renal function through its downstream attenuation of inflammatory pathway (TLR-4/NF-κB/TNF-α/IL-6 axis), apoptotic pathway (NF-κB/miRNA-375/HNF-1B/caspase-3 axis) and fibrotic pathway (TGF-β1/TNF-α axis). This inhibitory effect of SFZ on NF-κB could be at least in part attributed to its upstream modulation of p-AMPK/NF-κB axis, miRNA-132-3p/SIRT-1/NF-κB axis and SIRT1/miRNA-375/NF-κB axis. The mechanistic graphical presentation summarizes these effects of sulfasalazine on cisplatin-induced nephrotoxicity (Fig. 9). This paves the way to further future preclinical/clinical studies to elucidate other functional and molecular mechanisms to document potential beneficial therapeutic outcomes of using sulfasalazine, not only, in the context of chronic cisplatin-induced nephrotoxicity, but also in tumor-bearing models to evaluate the potential effects of sulfasalazine on antitumor effects of Cisplatin.

## Supplementary Information

Below is the link to the electronic supplementary material.

ESM1 (XLSX 12.9 KB)

ESM2 (XLSX 9.28 KB)

## Data Availability

All data generated or analyzed during this study are included in this published article.

## Abbreviations

Cis: Cisplatin
SFZ: Sulfasalazine
pNF-κB: Phosphorylated nuclear factor-kappa B
TNF-α: Tumor necrosis factor-alpha
TGF-β1: Transforming growth factor-beta1
IL-1: Interleukin-1
IL-6: Interleukin-6
TLR-4: Toll-like receptor 4
DAMPs: Damage associated molecular patterns
HNF-1β: Hepatocyte nuclear factor-1 beta
p-AMPK: Phosphorylated AMP-activated protein kinase
SIRT-1: Sirtuin 1
TAC: Total Anti-oxidant Capacity
